# *Lactiplantibacillus plantarum* GUANKE alleviates Zearalenone-induced intestinal dysfunction by modulating oxidative stress and inflammation

**DOI:** 10.1371/journal.pone.0351300

**Published:** 2026-07-01

**Authors:** Qiaoning Chang, Yanchao Zhao, Han Li, Bin Zhang, Meidi Zhang, Pei Zhao, Jingmin He, Bugao Li, Ming D. Li

**Affiliations:** 1 College of Animal Sciences, Shanxi Agricultural University, Taigu, Shanxi, China; 2 State Key Laboratory for Diagnosis and Treatment of Infectious Diseases, National Clinical Research Center for Infectious Diseases, Collaborative Innovation Center for Diagnosis and Treatment of Infectious Diseases, The First Affiliated Hospital, Zhejiang University School of Medicine, Hangzhou, China; 3 College of Biological Sciences, Shanxi Agricultural University, Taigu, Shanxi, China; Tanta University Faculty of Agriculture, EGYPT

## Abstract

**Aims:**

This study evaluated *L. plantarum* GUANKE’s protective effects on ZEN-induced intestinal dysfunction and explored its molecular mechanisms.

**Methods:**

In vitro, IPEC-J2 cells were divided into control, ZEN (40 μM), GUANKE (20 MOI), and ZEN+GUANKE groups (24 h treatment). Cell viability (CCK-8), oxidative stress indicators (LDH, ROS, T-SOD, GSH, MDA), and inflammatory cytokine mRNAs (*IL-1β, IL-6, TNF-α, IL-10*) were detected. In vivo, Balb/c mice were randomized into five groups (control, ZEN, GUANKE, ZEN + LG, ZEN + HG) for 28 days of intervention. Jejunum histology (H&E staining), oxidative stress/inflammatory factors (kits/ELISA), serum intestinal function indices (D-xylose, D-lactate, DAO), and transcriptomic analysis were performed.

**Results:**

*L. plantarum* GUANKE significantly improved IPEC-J2 cell viability, reduced LDH release (*P* < 0.01) and ROS accumulation *(P* < 0.001), restored T-SOD/GSH activities *(P* < 0.05), decreased MDA (*P* < 0.05), suppressed pro-inflammatory cytokines *(IL-1β, IL-6, TNF-α*; *P* < 0.05), and upregulated *IL-10* (*P* < 0.05). In mice*, L. plantarum* GUANKE increased villus height/crypt depth ratio (*P* < 0.01), restored antioxidant status (*P* < 0.05), and rebalanced cytokine expression (*P* < 0.05 for pro-inflammatory; *P* < 0.01 for IL-10). Transcriptomic analysis suggested that ZEN activated the NF-κB pathway, while *L. plantarum* GUANKE treatment was associated with reduced pathway activity.

## Introduction

Zearalenone (ZEN) is a nonsteroidal estrogenic mycotoxin produced by Fusarium species (a genus of filamentous fungi), and is frequently found in mold-contaminated grains such as corn, wheat, and barley [1]. As a toxic metabolite, ZEN exerts adverse effects on multiple organs in animals and humans [2]. ZEN also promotes ROS production, which in turn inhibits the activation of T and B lymphocytes through oxidative damage to signaling pathways and induction of mitochondrial dysfunction [3]. In the liver and kidney, ZEN induces marked effects, including fatty change, hepatitis, tubular degeneration, and necrosis [4,5]. Among these, intestinal dysfunction has emerged as a critical concern, as the intestinal mucosal epithelial cells are tightly interconnected, forming a robust barrier that effectively prevents most harmful substances and pathogens from entering the bloodstream [6]. This barrier serves as the primary physical defense against the absorption of toxins [7]. Previously reported studies have demonstrated that ZEN can disrupt the structural integrity of the intestinal epithelium and trigger inflammatory responses [8]. Given the significant impact of ZEN-induced damage on intestinal function, it is crucial to further investigate the underlying mechanisms of ZEN toxicity and develop effective strategies for its prevention and control [9].

Probiotics, defined as microorganisms that can colonize and proliferate in the gut while conferring health benefits to the host, have emerged as promising feed additives for mitigating the toxic effects of mycotoxins in animals [10]. For example, *Bacillus veles* A2 has been reported to alleviate ZEN-induced intestinal inflammation and tissue damage in the mouse cecum by regulating intestinal microbiota [11]. *Saccharomyces cerevisiae* has also been reported to attenuate the toxic effects of *Fusarium* toxins including ZEN [12]. In addition, certain probiotics have been shown to suppress pro-inflammatory cytokine expression and inhibit mycotoxin-induced inflammatory responses [13]. These findings support the contribution of probiotics in maintaining intestinal barrier integrity [14].

*Lactiplantibacillus plantarum* GUANKE (hereinafter referred to as *L. plantarum* GUANKE), a Gram-positive, facultative anaerobic rod-shaped bacterium isolated from healthy human feces, is an edible probiotic [15]. This strain exhibits high tolerance to bile acids and acidic environments, ensuring its survival and colonization in the intestine [15]. Previous studies have shown that *L. plantarum* GUANKE possesses immunomodulatory properties, including the ability to upregulate the interferon signaling pathway and to promote T and B lymphocyte activity [16], as well as anti-inflammatory effects through modulation of cytokines and chemokines [17]. However, whether *L. plantarum* GUANKE could protect against ZEN-induced intestinal injury remains unknown. Therefore, this study sought to address this gap by systematically evaluating the protective effect of *L. plantarum* GUANKE against ZEN-induced intestinal dysfunction and elucidated its underlying molecular mechanisms.

## Materials and methods

### Drug, probiotics and cell culture

The Zearalenone used in this report was purchased from Sichuan Jingcui Tiancheng Pharmaceutical Technology Co., Ltd., with a purity of > 98%. The *L. plantarum* GUANKE utilized in this study was a laboratory-maintained strain, and its identity was previously rigorously confirmed via whole-genome sequencing (WGS) [18]. IPEC-J2 cells utilized in this research were obtained from the Shanghai Cell Bank of the Chinese Academy of Sciences. All abbreviations and the experimental procedure used in this report are given in S1 Table and S1 Fig, respectively.

### Ethics statement

All experimental procedures involving animals were reviewed and approved by the Institutional Animal Care and Use Committee (IACUC) of the First Affiliated Hospital of Zhejiang University School of Medicine (Approval #: 2023-1374). All methods were carried out in accordance with the National Institutes of Health Guide for the Care and Use of Laboratory Animals and in compliance with relevant institutional, provincial, and federal regulations.

### Assessment of cell viability and cytotoxicity

**Fig 1:**
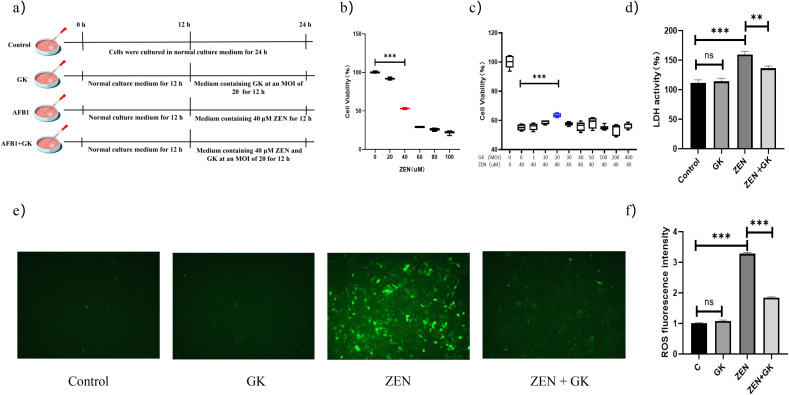
Protective effects of *L. plantarum* GUANKE on ZEN (Zearalenone)-induced cytotoxicity in IPEC-J2 cells. (a) Cell Experiment. (b) Viability of IPEC-J2 cells treated with different concentrations of ZEN. (c) Cell viability detected after 12 hours of treatment with 40μM ZEN and *L. plantarum* GUANKE at MOI (multiplicity of infection) of 20:1 (data expressed as Mean ± SEM; n = 5). (d) LDH (lactate dehydrogenase) activity detected in cells treated with 40μM ZEN, *L. plantarum* GUANKE at MOI = 20:1, or both for 12 hours. (e, f) ROS (reactive oxygen species) levels measured in cells treated with 40μM ZEN, *L. plantarum* GUANKE at MOI of 20:1, or both for 12 hours (data expressed as Mean ± SEM; n = 3).

IPEC-J2 cells were seeded into 96- or 6-well plates depending on the assay requirements. The detailed cell grouping and treatment protocols are illustrated in Fig 1a. To establish the optimal treatment parameters, preliminary cell viability screenings were conducted using a Cell Counting Kit-8 (CCK-8) assay (Beyotime Biotechnology, Shanghai, China). Cells were firstly exposed to a range of ZEN concentrations (0–100 μM) for 12 h, which identified 40 μM as the optimal modeling dose based on the half-maximal inhibitory concentration (IC50). Secondly, co-treatment with 40 μM ZEN and varying multiplicities of infection (MOIs) of *L. plantarum* GUANKE established 20:1 as the optimal bacteria-to-cell protective ratio. For the assessment of cell viability in these assays, 10 μL of CCK-8 solution was added to each well. Following a 2-h incubation in the dark at 37°C, the absorbance was measured at 450 nm using a microplate reader (Thermo Fisher Scientific, USA).

For cytotoxicity assessment, culture supernatants were collected and centrifuged at 4000 rpm for 5 min. Then, 120 μL of the supernatant was mixed with 60 μL of working solution from a lactate dehydrogenase (LDH) cytotoxicity detection kit (Beyotime Biotechnology, C0018S). Following a 30 min incubation in the dark at 37°C, absorbance was measured at 490 nm to quantify LDH release.

### Measurement of intracellular reactive oxygen species (ROS) accumulation

IPEC-J2 cells were seeded in 6-well plates (1 × 10^6^ cells/well) and treated as designated at 60–70% confluency. Intracellular ROS levels were measured using a commercial ROS assay kit (Beyotime Biotechnology, S0033S, China) based on the fluorescent probe 2′,7′-dichlorodihydrofluorescein diacetate (DCFH-DA). Cells were washed with PBS, incubated with 10 μM DCFH‑DA at 37°C for 20 min in the dark and washed again. Subsequently, fluorescence was quantified at 488/525 nm (ex/em) using a fluorescence microplate reader. Representative images were acquired with a fluorescence microscope (Leica, Germany).

### Evaluation of intracellular antioxidant capacity and lipid peroxidation

IPEC-J2 cells were seeded in 6‑well plates (1 × 10^6^ cells/well). After treatment, cells were lysed in RIPA buffer (Beyotime Biotechnology), and protein concentrations were determined using a BCA kit (Beyotime Biotechnology, P0009). Oxidative stress indicators were measured as follows: reduced glutathione (GSH) with a GSH assay kit (Nanjing Jiancheng Bioengineering Institute, A006‑2‑1; absorbance at 405 nm), malondialdehyde (MDA) with an MDA colorimetric kit (Elabscience, E‑BC‑K028‑M; absorbance at 532 nm), and total superoxide dismutase (T‑SOD) activity with a T‑SOD colorimetric kit (Elabscience, E‑BC‑K020‑M; absorbance at 450 nm). Levels of GSH, MDA, and T‑SOD were calculated as suggested by the manufacturer and normalized to cellular protein content.

### Gene expression analysis via quantitative real-time PCR (qRT-PCR)

Total RNA was extracted from IPEC-J2 cells using the Cell/Tissue MiRNA Extraction Kit (Vazyme Biotech, RC201) according to the manufacturer’s instructions. The concentration and purity of the extracted RNA were measured using a NanoDrop 2000 Spectrophotometer (Thermo Fisher Scientific, USA). The RNA was then reverse transcribed into complementary DNA (cDNA) using Reverse Transcription Kit (Vazyme Biotech, RL201-01). Quantitative real-time PCR (qRT-PCR) was carried out using a SYBR Green Master Mix on a 7500 Real-Time PCR System (Applied Biosystems, USA). The thermal cycling conditions consisted of an initial denaturation at 95°C for 10 min, followed by 40 cycles of amplification (95°C for 10 s and 60°C for 1 min). The specific primers employed in this experiment are listed in S2 Table. The relative mRNA expression levels of the target genes were calculated using the 2^—ΔΔCt^ method, with *GAPDH* serving as the endogenous reference gene for normalization.

### Animal experiments design and treatment

Fifty male BALB/c mice (7–8 weeks old) were obtained from Jiangsu Jicui Yaokang Biotech Co. (GemPharmatech Co., Ltd., China) and acclimated for one week before the experiment. The mice were randomly divided into five groups (n = 10 per group). The experimental design is summarized in Fig 3a. The ZEN dose (40 mg/kg) was selected based on the literature [6,11], and the doses of *L. plantarum* GUANKE (10⁶ and 10¹⁰ CFU) were based on a previous study [19]. No mortality occurred during the 28-day study. All tissues collection procedures were performed under sodium pentobarbital anesthesia with measures to minimize animal suffering.

### Sample collection and preparation

During the 28‑day trial, mouse behavior, feed intake, and water consumption were monitored daily, and body weight were measured regularly. After the last day of treatment, animals were fasted for 16 h, weighed, and anesthetized via intraperitoneal sodium pentobarbital injection (50 mg/kg body weight). Blood samples were collected, allowed to clot, and centrifuged (3000 × g, 20 min, 4°C) to obtain serum. Following euthanasia, jejunal tissues were rapidly excised, rinsed with ice‑cold saline, and divided into three parts for: (1) fixation in 4% paraformaldehyde for histopathology; (2) homogenization for oxidative‑stress and cytokine assays; and (3) snap-freezing in liquid nitrogen and stored at −80°C for molecular analysis.

### Histopathological analysis via H&E staining

Following a 24-hour fixation period in 4% paraformaldehyde, the jejunum samples underwent meticulous tissue trimming and was subsequently processed through a series of standard histological procedures. This included gradient alcohol dehydration, xylene clearing (xylene I and II, 45 min each) and wax infiltration in molten paraffin at 60°C (twice, 2 h each). The tissue was then sectioned into 5-μm-thick slices using a Leica RM2235 rotary microtome. The sections were deparaffinized in xylene (10 min, twice), followed by conventional staining with Harris hematoxylin for 5 min and eosin for 2 min, and finally mounted with neutral resin [6]. Images were captured using a microscope system, with each sample being photographed under a 20x microscope objective to assess the structural morphology of the jejunum.

To explicitly address ZEN-induced morphological alterations, software-assisted quantification was employed to evaluate villus atrophy and crypt hyperplasia. For each sample, at least 10 well-oriented, intact villus-crypt units were randomly selected. The absolute villus height (VH) and crypt depth (CD) were precisely measured using Image-Pro Plus 6.0 software, and the VH-to-CD ratio (V/C ratio) was subsequently calculated. Furthermore, to provide standardized criteria for local pathological lesions, histological scoring was performed blindly by two independent observers based on the Ulrike Erben scoring system [20]. The detailed scoring criteria for parameters such as inflammatory cell infiltration and epithelial hyperplasia are outlined in Table 1.

**Table 1:** Criteria for semiquantitative histopathological scoring of jejunal sections (H&E staining).

Category	Criterion	Definition	Score
Inflammatory cell infiltrate	Severity	Minimal: < 10%	1
		Mild: 10–25%; scattered neutrophils	2
		Moderate: 26–50%	3
		Marked: > 51%; dense infiltrate	4
Epithelial changes	Hyperplasia	Minimal: < 25%	1
		Mild: 25–35%	2
		Moderate: 36–50%; mitoses in middle/upper third of crypt epithelium, distant from crypt base	3
		Marked: > 51%; mitoses in upper third of crypt epithelium, distant from crypt base	4
Mucosal architecture	Villous blunting	Mild: villous-to-crypt-length ratio of 2:1 to 3:1	1–3
		Moderate: villous-to-crypt-length ratio of 1:1 to 2:1	2–4
		Villous atrophy	3–5

### Determination of intestinal permeability

Serum samples were used to assess intestinal permeability and mucosal integrity by measuring D‑xylose, diamine oxidase (DAO), and D‑lactic acid levels with commercial kits. D‑xylose concentration was measured with a D‑xylose assay kit (Nanjing Jiancheng Bioengineering Institute, A035‑1‑1) and the absorbance was read at 554 nm. Serum DAO activity was measured with a DAO test kit (Nanjing Jiancheng Bioengineering Institute, A088‑3‑1) and the absorbance was recorded at 340 nm. D‑lactic acid content was determined with a D‑lactic acid assay kit (Jiangsu Aidison Biotechnology Co, ADS‑W‑T017) and the absorbance was measured at 340 nm. Final concentrations were calculated according to the kit protocols.

### Measurement of intestinal oxidative stress and inflammatory cytokines

Jejunal tissue samples were homogenized in cold RIPA lysis buffer (Beyotime Biotechnology, China). After centrifugation at 12,000 × g for 15 minutes at 4°C, the supernatants were collected. The total protein concentration of the tissue homogenates was determined using a BCA protein concentration assay kit (Beyotime Biotechnology, P0009) to normalize all subsequent biochemical measurements.

Oxidative stress markers in jejunal homogenates were measured with commercial kits: reduced glutathione (GSH) using a GSH assay kit (Nanjing Jiancheng Bioengineering Institute, A006‑2‑1; absorbance at 405 nm), malondialdehyde (MDA) with an MDA colorimetric kit (Nanjing Jiancheng Bioengineering Institute, A003‑1‑2; absorbance at 532 nm), and total superoxide dismutase (T‑SOD) activity with a T‑SOD colorimetric kit (Elabscience, E‑BC‑K020‑M; absorbance at 450 nm).

Inflammatory cytokines (IL‑1β, IL‑6, IL‑10, TNF‑α) were quantified with high‑sensitivity ELISA kits (Elabscience, E‑HSEL‑M0001, E‑HSEL‑M0003, E‑HSEL‑M0004, E‑HSEL‑M0009) using a sandwich enzyme immunoassay; absorbance was read at 450 nm. The actual levels of oxidative stress indicators and inflammatory cytokines were calculated based on the standard curves or formulas provided in the respective kit instructions and normalized to the tissue protein concentration.

### RNA extraction, Library construction and sequencing

Total RNA was extracted from the jejunal tissues using a Cell/Tissue RNA Extraction Kit (Vazyme Biotech, RC201) according to the manufacturer’s instructions. To ensure the reliability of the sequencing data, the concentration, purity, and integrity of the extracted RNA were strictly evaluated using a spectrophotometer and a bioanalyzer system. Only highly qualified RNA samples were utilized for downstream library construction.

Sequencing libraries were generated following standard protocols for Illumina platforms. mRNA was purified from the total RNA using poly-T oligo-attached magnetic beads and subsequently fragmented. First-strand cDNA was synthesized using random hexamer primers, followed by second-strand cDNA synthesis. After adenylation of the 3’ ends and adapter ligation, the cDNA fragments were amplified via PCR to create the final cDNA libraries. The qualified libraries were pooled at equal molarity and sequenced on an Illumina platform to generate paired-end reads.

### Transcriptomic data processing and analysis

Raw sequencing data in FASTQ format were processed using fastp (v0.23.1) [21] to remove adapters and low-quality reads, yielding high-quality clean data. Principal Component Analysis (PCA) was performed utilizing the Novogene online platform (https://magic.novogene.com/). Differential expression analysis was conducted using the DESeq2 R package [22]. Differentially expressed genes (DEGs) were identified based on the thresholds of an adjusted P-value (Padj) < 0.05 and |log2(FoldChange)| > 0.26. Gene Set Enrichment Analysis (GSEA) and functional enrichment of DEGs, with a specific focus on immunity-associated pathways, were performed using the clusterProfiler [23] and Pathview [24] packages in R (v4.2.2). Pathways were considered significantly enriched when Padj < 0.05 and Normalized Enrichment Score (NES) > 1. All data visualizations were constructed using the ggplot2 package [25].

### Statistical analysis

The data were subjected to analysis and visualization using GraphPad Prism 8. The results are presented as the mean ± standard error of the mean (mean ± SEM). Statistical comparisons between the two groups were conducted using a two-tailed unpaired t-test. A p-value of less than 0.05 was deemed statistically significant.

## Results

### Protective effects of *L. plantarum* GUANKE on ZEN-induced cytotoxicity in IPEC-J2 Cells

Fig 1 shows the effect of *L. plantarum* GUANKE on ZEN-induced cytotoxicity in mouse IPEC-J2 cells, which were initially exposed to a range of ZEN concentrations (0, 10, 20, 40, 80, and 100 μM) for 12 hours, followed by an assessment of cell viability. The results showed a significant reduction in cell viability, with the IC50 observed at a ZEN concentration of 40 μM (Fig 1b, *P* < 0.001). Thus, 40 μM ZEN was selected as the treatment concentration for subsequent experiments. Based on this treatment model, various multiplicities of infection (MOI) of *L. plantarum* GUANKE were applied to assess its protective effect. As shown in Fig 1c, the highest cell viability was observed at an MOI of 20:1 (bacteria-to-cell ratio), indicating an optimal protective concentration of *L. plantarum* GUANKE (*P* < 0.001).

To further evaluate the protective effect of *L. plantarum* GUANKE against ZEN-induced cellular damage, LDH release and intracellular ROS production were also assessed in IPEC-J2 cells. As shown in Fig 1d, ZEN exposure significantly increased LDH levels compared to the control group (*P* < 0.001). However, the ZEN + GK group markedly reduced LDH release (*P* < 0.01). Based on the ROS staining images (Fig 1e) and the ROS fluorescence intensity (Fig 1f), we observed an intense green fluorescence in the ZEN-treated group (*P* < 0.001), indicating a massive burst of reactive oxygen species (ROS). In contrast, this fluorescence intensity was markedly diminished in the ZEN + GK group (*P* < 0.001). These findings demonstrate that *L. plantarum* GUANKE protects against ZEN-induced cytotoxicity in IPEC-J2 cells by preserving cell viability, maintaining membrane integrity and suppressing early ROS accumulation.

### Ameliorative effects of *L. plantarum* GUANKE on ZEN-induced oxidative stress, inflammation, and apoptosis in IPEC-J2 cells

**Fig 2:**
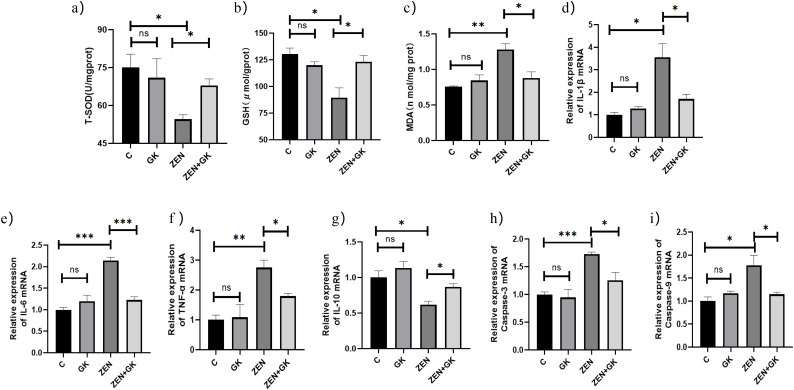
Ameliorative effects of *L. plantarum* GUANKE on ZEN (Zearalenone)-induced oxidative stress, inflammation, and apoptosis in IPEC-J2 cells. (a-c) Activities of T-SOD (total superoxide dismutase), GSH (glutathione), and MDA (malondialdehyde) detected in cells treated with 40μM ZEN, *L. plantarum* GUANKE at MOI (multiplicity of infection) of 20:1, or both for 12 hours. (d-i) mRNA expression levels of IL-1β (interleukin-1β), IL-6 (interleukin-6), TNF-α (tumor necrosis factor-α), IL-10 (interleukin-10), Caspase-3 and Caspase-9 genes detected in cells treated with 40μM ZEN, *L. plantarum* GUANKE at MOI of 20:1, or both for 12 hours (data expressed as Mean ± SEM; n = 3).

Given that *L. plantarum* GUANKE effectively suppressed the ZEN-induced ROS burst, we further investigated its regulatory effects on downstream antioxidant defense systems, inflammatory pathways, and apoptosis-related factors. As shown in Fig 2a-c, ZEN exposure significantly impaired cellular antioxidant capacity, evidenced by decreased T-SOD activity and GSH levels alongside elevated MDA content (T-SOD, *P* < 0.05; GSH, *P* < 0.05; MDA, *P* < 0.01), whereas co‑treatment with *L. plantarum* GUANKE restored T‑SOD and GSH while reducing MDA accumulation (all *P* < 0.05).

As indicated in Fig 2d-g, ZEN exposure triggered a pronounced inflammatory response, significantly upregulating the expression of pro-inflammatory cytokines (*IL-1β*, *P* < 0.05; *IL-6*, *P* < 0.001; *TNF-α*, *P* < 0.01) while suppressing the anti-inflammatory cytokine *IL-10* (*P* < 0.05). *L. plantarum* GUANKE intervention reduced these inflammatory mediators, significantly reducing the levels of *IL-1β* (*P* < 0.05), *IL-6* (*P* < 0.001), and *TNF-α* (*P* < 0.05), while increasing *IL-10* secretion (*P* < 0.05).

Additionally, ZEN exposure upregulated the expression of the pro-apoptotic proteins *Caspase-3* (*P* < 0.001) and *Caspase-9* (*P* < 0.05), the expression of which was significantly reduced by *L. plantarum* GUANKE treatment (Fig 2h-i, *P* < 0.05). Collectively, these results demonstrate that *L. plantarum* GUANKE exerts its potent cytoprotective effects against ZEN toxicity by simultaneously counteracting oxidative stress, resolving inflammation, and inhibiting apoptotic pathways.

### Protective effects of *L. plantarum* GUANKE on ZEN-induced growth retardation in Mice

**Fig 3:**
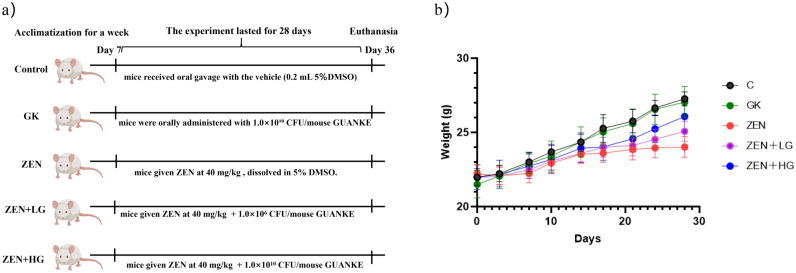
Protective effects of *L. plantarum* GUANKE on ZEN (Zearalenone)-induced growth retardation in mice. Control represents the control group, GK represents the *L. plantarum* GUANKE group, ZEN represents the zearalenone group, ZEN + LGK represents the low-dose *L. plantarum* GUANKE (10⁶ CFU) treatment group, and ZEN + HGK represents the high-dose *L. plantarum* GUANKE (10¹⁰ CFU) treatment group. (a) Animal Experiment. (b) Mouse Body Weight Change (data expressed as Mean ± SEM; n = 10).

To validate the protective effects of *L. plantarum* GUANKE in vivo, we established a ZEN-induced toxicity model in BALB/c mice (see Fig 3a for details). No abnormal mortality, behavioral lethargy, or severe signs of distress were observed across any of the experimental groups during the entire study period.

As shown in Fig 3b, Control and GK mice maintained healthy weight gain, whereas ZEN exposure severely suppressed growth. *L. plantarum* GUANKE alleviated this growth impairment in a dose-dependent manner, with the high-dose intervention restoring weight gain to levels close to the Control group, demonstrating its protective effect against ZEN-induced systemic toxicity.

### Restorative effects of *L. plantarum* GUANKE on ZEN-induced intestinal morphological damage in mice

**Fig 4:**
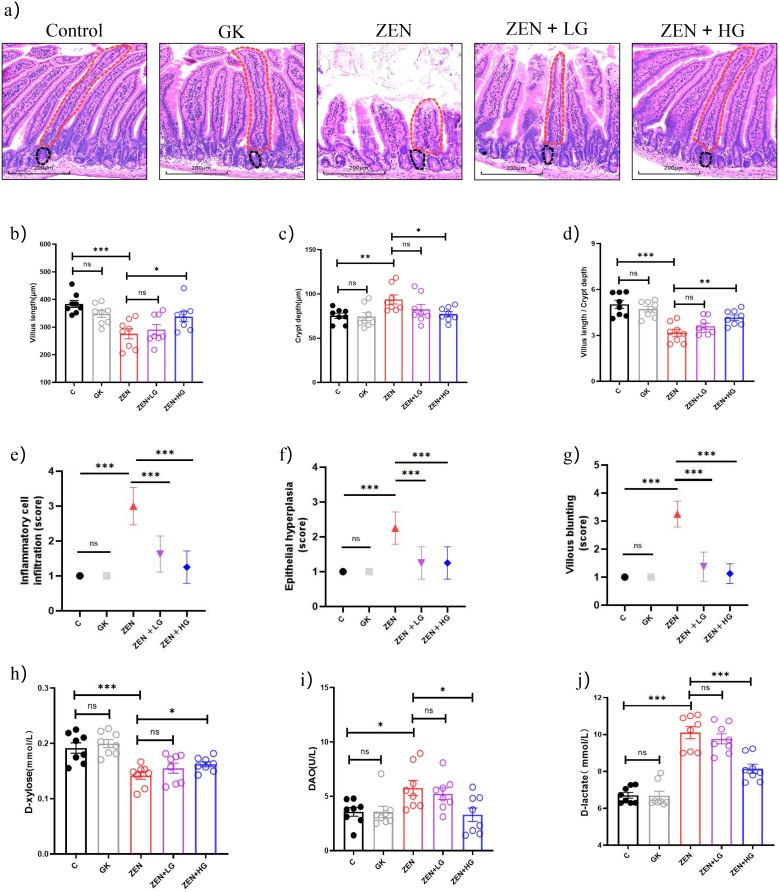
Restorative effects of *L. plantarum* GUANKE on ZEN (Zearalenone)-induced intestinal morphological damage in mice. (a) Observation of jejunum morphology by H&E (hematoxylin and eosin) staining (intestinal villi marked with red circles, crypts marked with black circles). (b-d) Measurement of villus height, crypt depth, and the ratio of villus length to crypt depth in the jejunum. (e-g) Scoring of inflammatory cell infiltrate, epithelial changes, and mucosal architecture in the jejunum. (h-j) Levels of D-xylose, DAO (diamine oxidase), and D-lactic acid in serum (data expressed as Mean ± SEM; n = 8).

Histopathological analysis revealed that ZEN exposure induced profound intestinal structural damage (Fig 4a). Specifically, ZEN significantly decreased villus length (Fig 4b, *P* < 0.001) and the villus-to-crypt ratio (Fig 4d, *P* < 0.001), while increasing crypt depth (Fig 4c, *P* < 0.01). Furthermore, ZEN triggered severe local lesions, as evidenced by drastically elevated pathological scores for inflammatory cell infiltration, epithelial hyperplasia, and villous blunting (Fig 4e-g, *P* < 0.001). Notably, both low and high doses of *L. plantarum* GUANKE effectively reduced these pathological lesion scores (*P* < 0.001). However, only the high-dose intervention (ZEN + HG) significantly restored the physical architecture, including villus length (*P* < 0.05), crypt depth (*P* < 0.05), and the villus-to-crypt ratio (*P* < 0.01).

Consistent with the morphological improvements, functional barrier integrity was also protected by *L. plantarum* GUANKE. ZEN exposure significantly increased severe intestinal permeability, characterized by reduced serum D-xylose absorption (Fig 4h, *P* < 0.001) and massive leakage of DAO (Fig 4i, *P* < 0.05) and D-lactate (Fig 4j, *P* < 0.001) into the bloodstream. High-dose *L. plantarum* GUANKE treatment successfully reversed these trends, significantly restoring D-xylose (*P* < 0.05) absorption and restricting the abnormal efflux of DAO (*P* < 0.05) and D-lactate (*P* < 0.001). Collectively, these findings demonstrate that high-dose *L. plantarum* GUANKE comprehensively protects against ZEN-induced intestinal injury by preserving physical architecture, suppressing pathological lesions, and maintaining functional permeability.

### Inhibitory effects of *L. plantarum* GUANKE on ZEN-induced oxidative stress and inflammatory responses in mice

**Fig 5:**
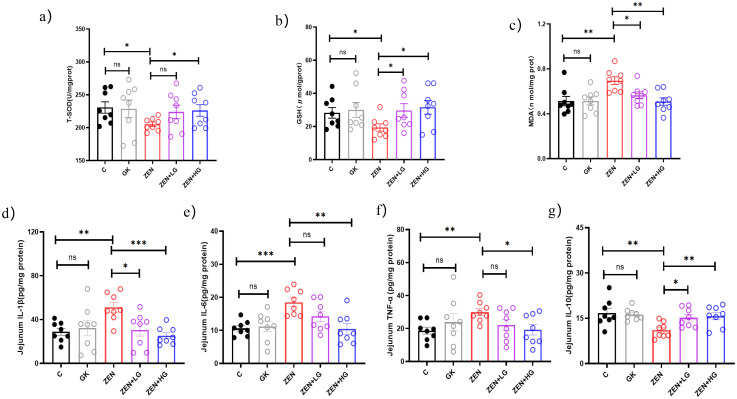
Inhibitory effects of *L. plantarum* GUANKE on ZEN (Zearalenone)-induced oxidative stress and inflammatory responses in mice. (a, b) Activities of antioxidant enzymes T-SOD (total superoxide dismutase) and GSH-Px (glutathione peroxidase) in the intestinal tract of mice. (c) Activity of MDA (malondialdehyde) in the intestinal tract of mice. (d-g) Secretion levels of pro-inflammatory factors IL-1β (interleukin-1β), IL-6 (interleukin-6), and TNF-α (tumor necrosis factor-α) in the intestinal tract of mice. (d) Secretion level of anti-inflammatory factor IL-10 (interleukin-10) in the intestinal tract of mice (data expressed as Mean ± SEM; n = 8).

The effects of *L. plantarum* GUANKE treatment on the oxidative stress and inflammation of mice exposed to ZEN are shown in Fig 5. Compared to the Control group (Fig 5a-5c), ZEN-treated mice exhibited significantly decreased T-SOD activity and GSH levels (all *P* < 0.05), alongside a marked increase in MDA accumulation (*P* < 0.01). However, *L. plantarum* GUANKE counteracted this oxidative injury. Specifically, GSH levels were effectively recovered across both intervention doses (*P* < 0.05), while T-SOD activity was improved exclusively by the high-dose treatment (*P* < 0.05). Concurrently, ZEN-induced lipid peroxidation was markedly mitigated, as evidenced by the reduction in MDA accumulation (ZEN + LG, *P* < 0.05; ZEN + HG, *P* < 0.01).

As indicated in Fig 5d-5g, *L. plantarum* GUANKE effectively resolved the inflammatory cascade. The expression of pro-inflammatory cytokines (IL-1β, IL-6, and TNF-α) was significantly inhibited (all *P* < 0.01), and the anti-inflammatory cytokine IL-10 was remarkably elevated (*P* < 0.01), primarily by high-dose *L. plantarum* GUANKE treatment (IL-1β, *P* < 0.001; IL-6, *P* < 0.01; TNF-*α*, P < 0.05; IL-10, P < 0.01).

### Immunomodulatory effects of *L. plantarum* GUANKE on ZEN-induced intestinal injury based on transcriptomic profiling

**Fig 6:**
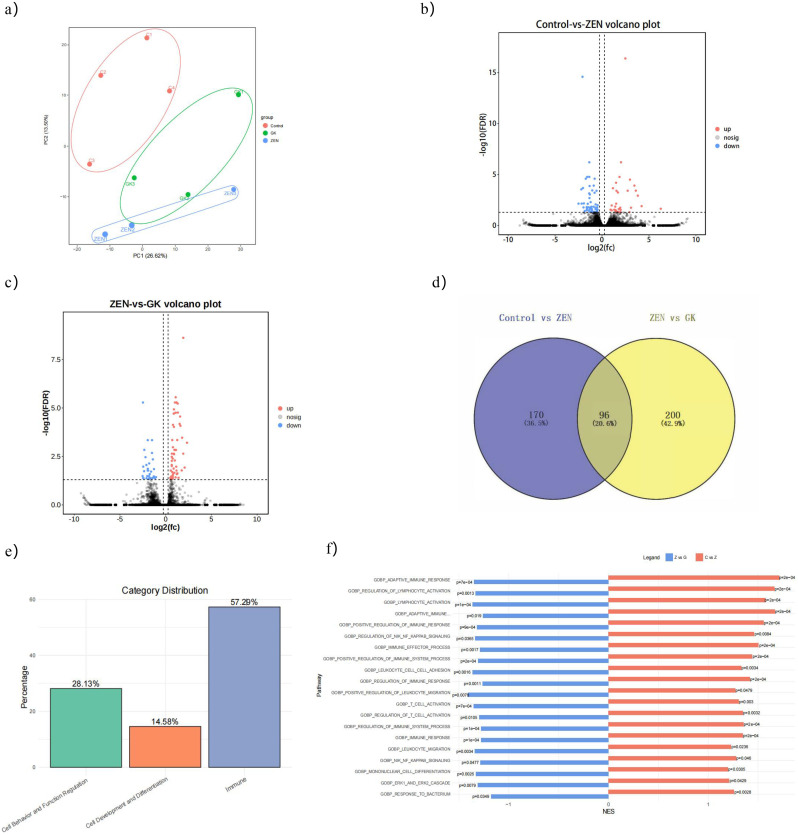
Immunomodulatory effects of *L. plantarum* GUANKE on ZEN (Zearalenone)-induced intestinal injury based on transcriptomic profiling. (a) Replicate correlation analysis of transcriptomic data. (b) Multidimensional scaling plot showing sample distribution (colored/shaped by experimental groups), where each point represents a gene. (c) Venn diagram of significantly enriched pathways *(P* < 0.05) from GSEA (Gene Set Enrichment Analysis) by comparing Control vs ZEN (266 pathways) and ZEN vs GK (296 pathways), with 96 overlapping pathways detected. (d) Functional classification of the 96 intersecting pathways. (e) Top 20 immunity-related pathways (prioritized by inflammatory factor association from ELISA (enzyme-linked immunosorbent assay) results) among the intersecting pathways, ranked by enrichment significance. ZEN represents the Zearalenone group, and GK represents the high-dose *L. plantarum* GUANKE (10¹⁰ CFU) treatment group..

Principal component analysis (PCA) revealed a clear separation between the Control and ZEN groups, indicating global transcriptomic alterations induced by ZEN exposure (Fig 6a). The transcriptomic profile of the *L. plantarum* GUANKE-treated group clustered closely with controls and shifted away from the ZEN group, suggesting an effective mitigation of ZEN-induced molecular perturbations. Differential expression analysis identified 86 differentially expressed genes (DEGs) in the ZEN group versus Controls (32 up, 54 down), while the *L. plantarum* GUANKE intervention modulated 90 DEGs compared to the ZEN group (59 up, 31 down) (Fig 6b-c; S3-S6 Tables). Among these, 11 key genes (e.g., *Csf1r, Cyp2c55, Itga4*) showed expression patterns that were reversed by *L. plantarum* GUANKE treatment. Functional annotation indicated that these core targets are primarily involved in immune regulation, energy metabolism, and detoxification.

Gene set enrichment analysis (GSEA) further elucidated the systemic mechanisms. A Venn diagram of enriched pathways showed 96 overlapping pathways (from 266 ZEN-altered and 296 GUANKE-modulated pathways) between the respective comparisons (Fig 6d). Predominant immune-related functions constituted 57.3% of this overlapping regulatory network (Fig 6e). Further focused examination of cytokine-associated pathways (e.g., *IL-1β, IL-6, IL-10, TNF-α*) revealed that the top 20 ZEN-upregulated immune pathways (such as adaptive immune response and NF-κB signaling) were consistently downregulated following *L. plantarum* GUANKE intervention (Fig 6f; S7 Table). These transcriptomic findings mechanistically support our biochemical data, demonstrating that the *L. plantarum* GUANKE alleviates ZEN-induced intestinal immunopathology through the multimodal regulation of immune networks.

## Discussion

Zearalenone is widely present in food and feed grains intended for human and animal consumption, posing a potential threat to international public health [26]. Accumulated evidence indicates that ZEN can reduce growth performance and impair animal health. This toxin primarily enters the animal body through contaminated feed, and the intestine is considered the first barrier against its harmful effects [8]. Therefore, in this study, IPEC-J2 cells and mice were selected as experimental models to investigate the intestinal toxicity of ZEN and to elucidate the alleviating effects and underlying mechanisms of *L. plantarum* GUANKE against zearalenone-induced intestinal injury.

We found that ZEN exposure significantly suppressed body weight gain in mice. After 28 days of treatment, the average body weight of ZEN-treated mice was approximately 27 g, whereas control mice reached 33 g. Notably, co-treatment with high-dose *L. plantarum* GUANKE (10¹⁰ CFU) effectively attenuated ZEN-induced weight loss, with body weight reaching approximately 32 g, similar to the control group. The low-dose *L. plantarum* GUANKE (10^6^ CFU) showed only a modest effect. These results indicate that ZEN induces systemic toxicity manifested as reduced growth performance, and *L. plantarum* GUANKE, particularly at the high dose, protects against this effect. This finding is consistent with previous reports showing that ZEN exposure impairs body weight gain in rodents, and further supports the protective effect of *L. plantarum* GUANKE against ZEN-induced systemic toxicity.

The integrity of the intestinal architecture is fundamental for maintaining the functional capacity of the intestinal barrier [6]. Upon entry into the host, ZEN primarily targets intestinal epithelial cells and disrupts cellular structure and function. Our results demonstrate that ZEN significantly reduces cell viability and increases LDH release, consistent with previous findings that ZEN exerts its direct cytotoxic effects leading to apoptosis [27,28]. Notably, administration of *L. plantarum* GUANKE substantially restored cell viability and reduced LDH release, indicating a protective effect against ZEN-induced cytotoxicity. These findings are in agreement with a recent study showing that engineered probiotics expressing lactonase ZHD101 effectively alleviated ZEN-induced intestinal disruption in rats [29].

ZEN-induced structural alterations in the intestine are typically characterized by reductions in villus height, villus blunting, increases in crypt depth, and a decreased villus-to-crypt ratio, all of which are important indicators of intestinal absorptive capacity [30]. These morphological changes have been consistently reported in piglets and laying hens, and are known to impair nutrient absorption and weaken disease resistance [31,32]. Histological analyses via H&E staining revealed that ZEN treatment induced detectable villus shortening, villus blunting, crypt hyperplasia and inflammatory infiltration, leading to a statistically significant reduction in the villus-to-crypt ratio and an increased histological score in the jejunum of mice. Although these morphological alterations were relatively modest, the changes in the villus-to-crypt ratio reached statistical significance, supporting the protective effect of *L. plantarum* GUANKE. Consistent with our findings, previous studies have demonstrated that probiotic administration can ameliorate ZEN-induced intestinal morphological damage [29]

The protective effects of *L. plantarum* GUANKE observed in IPEC-J2 cells were consistently reproduced in the mouse model. Both in vitro and in vivo experiments demonstrated that *L. plantarum* GUANKE alleviated ZEN-induced oxidative stress, suppressed inflammatory responses, and improved intestinal barrier integrity. It should be noted, however, that most of these changes did not fully return to the control levels. This consistency is not surprising, as IPEC-J2 cells were derived from porcine jejunal epithelium and represent the primary target cells of ZEN in the intestine [33,34]. Thus, the cellular responses observed in vitro likely reflect the key mechanisms operating in the complex in vivo setting [34], where *L. plantarum* GUANKE similarly acts on intestinal epithelial cells to enhance antioxidant and anti-inflammatory defenses.

Increased intestinal permeability is another hallmark of barrier dysfunction. Serum biomarkers such as DAO, D-lactic acid, and D-xylose are commonly used to evaluate permeability [40]. DAO is an intracellular enzyme primarily localized in the villus epithelial cells of the intestinal mucosa. Under normal conditions, DAO activity in circulation is very low; however, when the intestinal mucosal barrier is damaged, DAO is released into the bloodstream, making it a sensitive indicator of mucosal structural integrity [35]. D-lactic acid is a metabolite produced by bacterial fermentation in the intestinal lumen. Mammals lack the enzyme system to efficiently metabolize D-lactate, so elevated plasma D-lactate levels directly reflect increased intestinal permeability and bacterial translocation across the damaged mucosal barrier [36]. D-xylose, in contrast, is a pentose sugar that is absorbed passively in the small intestine. A decrease in serum D-xylose levels indicates impaired intestinal absorptive capacity, which is a functional consequence of mucosal damage [37]. Thus, these three markers complement each other: DAO reflects structural damage to the intestinal epithelium, D-lactate indicates permeability changes and bacterial translocation, and D-xylose assesses absorptive function. The combination of these markers provides a more complete picture of intestinal barrier status than any single marker alone [38]. Our findings showed that *L. plantarum* GUANKE administration significantly reduced DAO and D-lactic acid levels while increasing D-xylose concentrations in ZEN-treated mice, indicating improved barrier integrity and absorptive function. Consistent with our results, a recent study reported that ZEN exposure significantly decreased plasma D-xylose levels while increasing D-lactate and DAO activities in piglets, and that dietary intervention reversed these changes [30].

ZEN is also known to provoke oxidative stress, leading to the depletion of key antioxidant defenses, including T-SOD and GSH, and to increased levels of MDA, a marker of lipid peroxidation [39,40]. Previous studies have confirmed that ZEN exposure can induce excessive accumulation of reactive ROS and elevated MDA levels in mice, while reducing GSH levels and inhibiting the activities of SOD and CAT [41]. Similar changes have also been observed in aquatic animal models such as zebrafish embryos: ZEN treatment similarly promotes ROS production, decreases the activities of SOD, GSH-Px and CAT, and reduces GSH content [42]. Our both in vitro and in vivo results consistently showed that ZEN exposure led to decreased T-SOD and GSH activity and elevated MDA levels, whereas *L. plantarum* GUANKE treatment reversed these trends. Elevated levels of ROS, which result from oxidative damage, were also observed following ZEN exposure and were significantly diminished by *L. plantarum* GUANKE, reinforcing its antioxidative role. These findings are consistent with recent studies indicating that *Lactobacillus* strains can alleviate ZEN-induced oxidative stress [43,44]. For example, *Bacillus velezensis* A2 has been reported to ameliorate ZEN-induced oxidative stress in IPEC-J2 cells by reducing ROS and MDA levels and restoring antioxidant enzyme activities [45].

Beyond oxidative damage, ZEN can also induce excessive secretion of pro-inflammatory cytokines by intestinal epithelial cells, thereby promoting inflammation. Consistent with the literature, our data demonstrated that ZEN upregulated the expression of IL-1β, IL-6 and TNF-α, but downregulated the anti-inflammatory cytokine IL-10 [45,46]. These findings are in line with previous studies in which similar cytokine dysregulation was observed in ZEN-exposed intestinal and immune cells [47,48]. *L. plantarum* GUANKE treatment reversed these cytokine alterations both in vitro and in vivo. Furthermore, ZEN-induced apoptosis is mediated through the mitochondrial (intrinsic) pathway, as evidenced by the upregulation of Caspase-9 (an initiator caspase specific to the mitochondrial pathway) and its downstream effector Caspase-3, as detected by qRT-PCR [49]. As expected, ZEN significantly upregulated the expression of both caspases, while *L. plantarum* GUANKE treatment reduced their expression, suggesting an anti-apoptotic effect in ZEN-induced intestinal toxicity. This aligns with the established understanding that activation of the mitochondrial pathway plays a critical role in ZEN-induced programmed cell death.

Although the protective effects of *L. plantarum* GUANKE against ZEN toxicity were evident in both cell and animal models, the underlying molecular mechanisms warranted a deeper exploration. Transcriptomic analysis, was employed to elucidate these mechanisms. Our RNA-sequencing analysis of jejunal tissues compared the Control, ZEN, and GK (*L. plantarum* GUANKE + ZEN, high-dose) groups. This analysis revealed that *L. plantarum* GUANKE treatment modulated the expression of immune-related genes (e.g., *Csf1r, Itga4, Mpeg1, Ndufa3, Leap2, mt-Tn, mt-Tc* and *mt-Tl2*) and immune-related pathways, most notably the nuclear factor kappa-B (NF-κB) signaling pathway.

Among these genes, several are functionally linked to the NF-κB signaling pathway and may contribute to intestinal barrier protection through this pathway. *Csf1r* is essential for the differentiation and function of macrophages, which are critical for intestinal immune surveillance and barrier maintenance [50]. Notably, *Csf1r* acts as an upstream activator of NF-κB signaling; thus, *L. plantarum* GUANKE-mediated restoration of *Csf1r* expression may suppress ZEN-induced NF-κB activation. *Leap2* is an antimicrobial peptide that directly inhibits bacterial growth and modulates gut microbiota composition [51]. Given that *Leap2* expression is regulated by the NF-κB pathway, its upregulation by *L. plantarum* GUANKE may enhance local antimicrobial defense and reduce inflammation. *Itga4* is directly involved in NF-κB signaling and its modulation by *L. plantarum* GUANKE may further contribute to immune regulation and barrier protection [52,53]. Collectively, the modulation of these NF-κB-linked genes by *L. plantarum* GUANKE likely contributes to the suppression of ZEN-induced inflammatory responses.

In addition to the immune-related genes discussed above, our transcriptomic analysis also identified changes in several genes involved in mitochondrial function and energy metabolism, including *Ndufa3, mt-Tn, mt-Tc,* and *mt-Tl2*. ZEN exposure led to the downregulation of these genes, while *L. plantarum* GUANKE treatment restored their expression. Mitochondria are essential for ATP production, and adequate energy supply is critical for intestinal epithelial wound healing [54]. Conversely, mitochondrial dysfunction is a well-established driver of intestinal epithelial damage and barrier disruption [55]. Based on these observations, we speculate that ZEN-induced downregulation of these genes may impair mitochondrial function [56] and ATP production, thereby compromising epithelial repair, while *L. plantarum* GUANKE-mediated restoration may improve mitochondrial energy metabolism and support intestinal epithelial repair. We acknowledge that direct evidence linking these specific genes to intestinal repair is limited, and future studies are needed to validate this hypothesis.

Furthermore, mycotoxins such as ZEN and DON can activate the NF-κB pathway, leading to the overexpression of IL-1β, IL-6, and TNF-α, and triggering a positive feedback loop via ROS-mediated NF-κB activation [57]. Consistent with this, our transcriptomic data indicate that ZEN activates the NF-κB pathway, in agreement these previous reports. Although direct evidence linking probiotics to NF-κB suppression has been limited, our study suggests that *L. plantarum* GUANKE may attenuate ZEN-induced oxidative and inflammatory responses through NF-κB pathway modulation.

Based on the collective evidence, we propose a working model for *L. plantarum* GUANKE’s protective mechanism against ZEN-induced intestinal injury. *L. plantarum* GUANKE appears to act primarily by enhancing the host’s endogenous defense systems rather than by directly neutralizing ZEN. Specifically, *L. plantarum* GUANKE alleviates oxidative stress and suppresses inflammation. Transcriptomic analysis suggested that these effects are associated with modulation of the NF-κB signaling pathway. We speculate that *L. plantarum* GUANKE may interfere with ZEN-induced NF-κB activation, thereby breaking the vicious cycle of ROS production and inflammatory cytokine release. Whether *L. plantarum* GUANKE can also directly bind or degrade ZEN remains unknown. We acknowledge that direct evidence for NF-κB involvement (e.g., p65 phosphorylation or IκBα degradation) was not obtained, and further studies are needed. Additionally, the role of gut microbiota was not investigated. Given that Lactobacillus strains can modulate gut microbiota and enhance barrier integrity [58], and ZEN exposure disrupts gut microbial balance [8], it is plausible that *L. plantarum* GUANKE may also exert its protective effects, at least in part, by stabilizing gut microbial communities. Future studies, including 16S rRNA sequencing of fecal or cecal samples, are warranted to test this hypothesis. Furthermore, villus width and goblet cell count were not assessed in this study. These parameters are known to reflect intestinal health and barrier function [59]. Future studies should include them for a more comprehensive evaluation. Finally, the transcriptomic analysis was performed only on the Control, ZEN, and high-dose GK (ZEN + 10¹⁰ CFU) groups. The low-dose treatment group (ZEN + LG) was not included due to the concerns of its value, cost of RNA-seq analysis as well as the exploratory nature of this analysis, which prioritized the high-dose intervention where the protective effects were most pronounced.

## Conclusion

In sum, our study demonstrates that *L. plantarum* GUANKE effectively protects against ZEN-induced intestinal dysfunction through multiple mechanisms, including reducing oxidative stress, suppressing inflammation, and preserving intestinal barrier integrity. Transcriptomic analysis suggested that these protective effects may be associated with modulation of the NF-κB pathway. Notably, the high-dose *L. plantarum* GUANKE (10¹⁰ CFU) showed superior protective effects compared to the low-dose regimen. Together, these findings support *L. plantarum* GUANKE as a promising probiotic candidate for mitigating ZEN-induced intestinal injury. Building on these findings, future studies should focus on validating the molecular mechanisms at the protein level, exploring the role of gut microbiota, and evaluating the potential application of *L. plantarum* GUANKE as a feed additive in animal husbandry.

## Supporting information

S1 Fig Experimental outline and procedure used in the study. (PDF)

S1 Table Abbreviations. (DOCX)

S2 Table Primer sequences. (DOCX)

S3 Table Upregulated differentially expressed genes (DEGs) in ZEN group compared with control group. (DOCX)

S4 Table Downregulated differentially expressed genes (DEGs) in ZEN group compared with control group. (DOCX)

S5 Table Upregulated differentially expressed genes (DEGs) in GK group vs. ZEN group. (DOCX)

S6 Table Downregulated differentially expressed genes (DEGs) in GK group vs. ZEN group. (DOCX)

S7 Table GSEA enrichment analysis. (DOCX)
