# Supplementary material for: Lactiplantibacillus plantarum GUANKE alleviates Zearalenone-induced intestinal dysfunction by modulating oxidative stress and inflammation
Source: PLoS One. 2026 Jul 1;21(7):e0351300. doi: 10.1371/journal.pone.0351300 (PMC13322542; doi:10.1371/journal.pone.0351300)
Supplement: S1 Fig — (PDF) [file pone.0351300.s001.pdf]

Lactiplantibacillus plantarum GUANKE alleviates Zearalenone-induced intestinal dysfunction by modulating oxidative stress and inflammation

Research Background and Objectives:

- Feed ZEN pollution is severe, causing liver and kidney damage in animals
- Search for biological detoxification probiotics
- Candidate strain: *Lactobacillus plantarum* GUANKE
- Evaluate the efficacy of GUANKE in alleviating ZEN toxicity and its molecular mechanisms

Part 1: In Vitro Validation (IPEC-J2 Cell Model)

1. Determine ZEN and GUANKE treatment concentrations
2. Cell viability (CCK-8)、LDH release
3. ROS、 staining, antioxidant enzyme activities (T-SOD、 GSH、 MDA)
4. Inflammatory factor expression (mRNA: *IL-1β, IL-1β, TNF-α, IL-10*)
5. Programmed cell death expression (mRNA: *Caspase-3/9*)

Part 2: Animal Experiment (Mouse Intestinal Exposure Model)

1. H&E staining：Intestinal morphology、Villus length、Crypt dept、Villus/crypt ratio
2. Intestinal research：D - xylose、D - lactic acid、Diamine oxidase
3. Oxidative stress: MDA、T-SOD、GSH
4. Inflammatory response: IL-1β,TNF-α, IL-6 and IL-10

Part 3: Transcriptome sequencing.

1. RNA extraction.
2. Library construction.
3. Raw data quality control.
4. Data analysis.
