# Supplementary material for: Lactiplantibacillus plantarum GUANKE alleviates Zearalenone-induced intestinal dysfunction by modulating oxidative stress and inflammation
Source: PLoS One. 2026 Jul 1;21(7):e0351300. doi: 10.1371/journal.pone.0351300 (PMC13322542; doi:10.1371/journal.pone.0351300)
Supplement: S1 Table — (DOCX) [file pone.0351300.s002.docx]

**S1 Table. Abbreviations**

| Abbreviation | Full name |
| --- | --- |
| ZEN | Zearalenone |
| *L. plantarum* GUANKE/GK | *Lactiplantibacillus plantarum* GUANKE |
| IPEC-J2 | Pig intestinal epithelial cell |
| MOI | multiplicity of infection |
| LDH | Lactate dehydrogenase |
| ROS | Reactive oxygen species |
| H&E | Hematoxylin and eosin |
| MDA | Malondialdehyde |
| T-SOD | Total Super Oxide Dismutase |
| GSH | Glutathione |
| IL-1β | Interleukin-1β |
| IL-6 | Interleukin-6 |
| IL-10 | Interleukin-10 |
| TNF-α | Tumour necrosis factor-α |
| Caspase-3 | Cysteinyl aspartate-specific proteinase 3 |
| Caspase-9 | Cysteinyl aspartate-specific proteinase 9 |
| DAO | Diamine oxidase |
| qRT-PCR | Quantitative Real-time polymerase chain reaction |
