# Supplementary material for: Lactiplantibacillus plantarum GUANKE alleviates Zearalenone-induced intestinal dysfunction by modulating oxidative stress and inflammation
Source: PLoS One. 2026 Jul 1;21(7):e0351300. doi: 10.1371/journal.pone.0351300 (PMC13322542; doi:10.1371/journal.pone.0351300)
Supplement: S2 Table — (DOCX) [file pone.0351300.s003.docx]

**S2 Table. Primer Sequences**

| Gene name | Accession numbers | Primer | GC% | Length (bp) |
| --- | --- | --- | --- | --- |
| *GAPDH* | NM_001206359.1 | F: ACATCATCCCTGCTTCTACTGG | 50.00 | 188 |
|  |  | R: CTCGGACGCCTGCTTCAC | 66.67 |  |
| *IL-1B* | NM_214055.1 | F: GCCAGTCTTCATTGTTCAGGTTTC | 45.83 | 74 |
|  |  | R: TAGCCATCACTTCCTTGGCG | 55.00 |  |
| *IL-6* | NM_214399.1 | F: TCTGGGTTCAATCAGGAGACC | 52.38 | 125 |
|  |  | R: ATCTGCACAGCCTCGACATT | 50.00 |  |
| *TNF-A* | NM_214022.1 | F: GGCCCAAGGACTCAGATCAT | 55.00 | 82 |
|  |  | R: CTGTCCCTCGGCTTTGACAT | 55.00 |  |
| *IL-10* | NM_214041.1 | F: CGGCCCAGTGAAGAGTTTCT | 55.00 | 98 |
|  |  | R: GGCAACCCAGGTAACCCTTA | 55.00 |  |
| *Caspase 3* | NM_214131.1 | F: CCGAGGCACAGAATTGGACT | 55.00 | 133 |
|  |  | R: TCGCCAGGAATAGTAACCAGG | 52.38 |  |
| *Caspase 9* | XM_003127618.4_ | F: AGCGCACTGGCTCCAATATC | 55.00 | 107 |
|  |  | R: CTGGACCATTTGCTTGGCAG | 55.00 |  |
