# Supplementary material for: Lactiplantibacillus plantarum GUANKE alleviates Zearalenone-induced intestinal dysfunction by modulating oxidative stress and inflammation
Source: PLoS One. 2026 Jul 1;21(7):e0351300. doi: 10.1371/journal.pone.0351300 (PMC13322542; doi:10.1371/journal.pone.0351300)
Supplement: S3 Table — (DOCX) [file pone.0351300.s004.docx]

**S3 Table. Upregulated differentially expressed genes (DEGs) in ZEN group compared with Control group**

| id | Gene name | Log_2_ Fold Change | FDR |
| --- | --- | --- | --- |
| ENSMUSG00000013643 | *Lypd8* | 0.9023 | 0.027565306 |
| ENSMUSG00000019772 | *Vip* | 2.4845 | 4.04287E-17 |
| ENSMUSG00000020866 | *Cacna1g* | 1.6632 | 0.046711007 |
| ENSMUSG00000022885 | *St6gal1* | 1.5044 | 0.007073224 |
| ENSMUSG00000023913 | *Pla2g7* | 1.5301 | 0.03892982 |
| ENSMUSG00000024621 | *Csf1r* | 1.4942 | 6.3109E-05 |
| ENSMUSG00000024747 | *Aldh1a7* | 1.0315 | 0.010662021 |
| ENSMUSG00000025002 | *Cyp2c55* | 2.9729 | 3.10045E-05 |
| ENSMUSG00000026421 | *Csrp1* | 1.4405 | 0.043977222 |
| ENSMUSG00000027009 | *Itga4* | 1.407 | 0.013744446 |
| ENSMUSG00000027797 | *Dclk1* | 1.4759 | 0.046418447 |
| ENSMUSG00000031673 | *Cdh11* | 1.9821 | 0.028716248 |
| ENSMUSG00000034258 | *Flvcr2* | 2.7322 | 0.000382619 |
| ENSMUSG00000036896 | *C1qc* | 1.6895 | 0.000567438 |
| ENSMUSG00000044583 | *Tlr7* | 2.92 | 0.048507516 |
| ENSMUSG00000046805 | *Mpeg1* | 1.5397 | 0.000387989 |
| ENSMUSG00000050621 | *Rps27rt* | 1.4354 | 0.029867928 |
| ENSMUSG00000051359 | *Ncald* | 1.3042 | 0.027829295 |
| ENSMUSG00000053279 | *Aldh1a1* | 1.1619 | 0.000215557 |
| ENSMUSG00000055730 | *Ces2a* | 1.8609 | 1.68562E-05 |
| ENSMUSG00000058135 | *Gstm1* | 1.0261 | 0.032192863 |
| ENSMUSG00000063286 | *Gvin-ps7* | 1.7608 | 0.044767036 |
| ENSMUSG00000066072 | *Cyp4a10* | 2.0217 | 6.16199E-07 |
| ENSMUSG00000072949 | *Acot1* | 1.9399 | 0.017712694 |
| ENSMUSG00000076540 | *Igkv4-80* | 6.2551 | 0.022193902 |
| ENSMUSG00000076934 | *Iglv1* | 1.9715 | 0.049534466 |
| ENSMUSG00000094006 | *Igkv4-59* | 3.0879 | 0.017712694 |
| ENSMUSG00000095351 | *Igkv3-2* | 3.5963 | 0.000377786 |
| ENSMUSG00000095630 | *Igkv6-23* | 3.8118 | 0.00116349 |
| ENSMUSG00000095753 | *Igkv4-53* | 4.2157 | 0.012450399 |
| ENSMUSG00000105606 | *Igkv2-109* | 3.4475 | 0.000119963 |
| ENSMUSG00000120754 | *Gm56536* | 1.785 | 0.023763266 |
