# Supplementary material for: Lactiplantibacillus plantarum GUANKE alleviates Zearalenone-induced intestinal dysfunction by modulating oxidative stress and inflammation
Source: PLoS One. 2026 Jul 1;21(7):e0351300. doi: 10.1371/journal.pone.0351300 (PMC13322542; doi:10.1371/journal.pone.0351300)
Supplement: S4 Table — (DOCX) [file pone.0351300.s005.docx]

**S4 Table. Downregulated differentially expressed genes (DEGs) in ZEN group compared with Control group**

| id | Gene name | Log_2_ Fold Change | FDR |
| --- | --- | --- | --- |
| ENSMUSG00000002228 | *Ppm1j* | -1.3237 | 0.000131883 |
| ENSMUSG00000003949 | *Hlf* | -1.63 | 0.017261686 |
| ENSMUSG00000017607 | *Tns4* | -0.7072 | 0.023763266 |
| ENSMUSG00000018500 | *Adora2b* | -1.0133 | 0.046418447 |
| ENSMUSG00000020988 | *L2hgdh* | -0.8874 | 0.000119963 |
| ENSMUSG00000021775 | *Nr1d2* | -0.5878 | 0.000387989 |
| ENSMUSG00000022389 | *Tef* | -1.0436 | 0.000357212 |
| ENSMUSG00000024521 | *Pmaip1* | -0.8213 | 0.046418447 |
| ENSMUSG00000027956 | *Tmem144* | -0.6701 | 0.017261686 |
| ENSMUSG00000028124 | *Gclm* | -0.6856 | 0.00835557 |
| ENSMUSG00000028737 | *Aldh4a1* | -1.2141 | 0.016538826 |
| ENSMUSG00000028957 | *Per3* | -1.3391 | 0.00201022 |
| ENSMUSG00000029186 | *Pi4k2b* | -0.5197 | 0.042356164 |
| ENSMUSG00000029352 | *Crybb3* | -1.7308 | 2.52272E-05 |
| ENSMUSG00000030834 | *Abcc6* | -0.7881 | 2.52272E-05 |
| ENSMUSG00000031618 | *Nr3c2* | -0.7351 | 0.045464296 |
| ENSMUSG00000032081 | *Apoc3* | -1.0272 | 0.03892982 |
| ENSMUSG00000032528 | *Vipr1* | -0.4713 | 0.027829295 |
| ENSMUSG00000032633 | *Flcn* | -0.5202 | 0.016525804 |
| ENSMUSG00000034413 | *Neurl1b* | -0.5316 | 0.046418447 |
| ENSMUSG00000034664 | *Itga2b* | -1.3572 | 1.68562E-05 |
| ENSMUSG00000035674 | *Ndufa3* | -0.467 | 0.009997536 |
| ENSMUSG00000036216 | *Leap2* | -1.2998 | 0.000896261 |
| ENSMUSG00000036466 | *Megf11* | -1.1243 | 0.014205915 |
| ENSMUSG00000036832 | *Lpar3* | -1.6462 | 0.031582271 |
| ENSMUSG00000037139 | *Myom3* | -1.0791 | 0.023763266 |
| ENSMUSG00000037613 | *Tnfrsf23* | -2.2018 | 0.000279302 |
| ENSMUSG00000038550 | *Ciart* | -2.0261 | 0.00020714 |
| ENSMUSG00000039096 | *Rsad1* | -1.2953 | 0.023763266 |
| ENSMUSG00000042684 | *Npl* | -1.6034 | 0.023763266 |
| ENSMUSG00000046516 | *Cox17* | -0.5246 | 0.031582271 |
| ENSMUSG00000049580 | *Tsku* | -0.7936 | 0.000602364 |
| ENSMUSG00000049690 | *Nckap5* | -1.1162 | 0.023763266 |
| ENSMUSG00000051811 | *Cox6b2* | -1.5197 | 0.007320739 |
| ENSMUSG00000052229 | *Gpr17* | -1.3657 | 0.000695049 |
| ENSMUSG00000053054 | *Adh6a* | -1.1122 | 0.031582271 |
| ENSMUSG00000055865 | *Tafa3* | -1.3301 | 0.043977222 |
| ENSMUSG00000055866 | *Per2* | -1.2112 | 0.005014402 |
| ENSMUSG00000059824 | *Dbp* | -2.0791 | 2.54822E-15 |
| ENSMUSG00000064348 | *mt-Tn* | -1.9373 | 0.006683724 |
| ENSMUSG00000064349 | *mt-Tc* | -1.405 | 0.033267272 |
| ENSMUSG00000064360 | *mt-Nd3* | -0.6219 | 0.023763266 |
| ENSMUSG00000064366 | *mt-Tl2* | -1.0995 | 0.045615238 |
| ENSMUSG00000068587 | *Mgam* | -0.5826 | 0.023763266 |
| ENSMUSG00000073102 | *Drc1* | -1.9767 | 0.016516908 |
| ENSMUSG00000074489 | *Bglap3* | -1.6266 | 0.015167189 |
| ENSMUSG00000079494 | *Nat8f5* | -1.5806 | 1.68562E-05 |
| ENSMUSG00000079495 | *Nat8f6* | -2.1735 | 0.007073224 |
| ENSMUSG00000093805 | *Gal3st2b* | -0.8909 | 0.014205915 |
| ENSMUSG00000104665 | *Gm43366* | -1.8523 | 0.037696204 |
| ENSMUSG00000109702 | *Gm45674* | -1.0979 | 0.028063513 |
| ENSMUSG00000109764 | *Klkb1* | -1.4314 | 0.016047725 |
| ENSMUSG00000114689 | *Gm9465* | -2.5029 | 0.007073224 |
| ENSMUSG00000120390 | *Gm56743* | -1.3574 | 6.16199E-07 |
