# Supplementary material for: Lactiplantibacillus plantarum GUANKE alleviates Zearalenone-induced intestinal dysfunction by modulating oxidative stress and inflammation
Source: PLoS One. 2026 Jul 1;21(7):e0351300. doi: 10.1371/journal.pone.0351300 (PMC13322542; doi:10.1371/journal.pone.0351300)
Supplement: S5 Table — (DOCX) [file pone.0351300.s006.docx]

**S5 Table. Upregulated differentially expressed genes (DEGs) in GK group vs. ZEN group**

| id | Gene name | Log_2_ Fold Change | FDR |
| --- | --- | --- | --- |
| ENSMUSG00000014294 | Ndufa2 | 0.6656 | 0.009846891 |
| ENSMUSG00000015656 | Hspa8 | 0.767 | 0.004058139 |
| ENSMUSG00000016252 | Atp5f1e | 0.9259 | 1.91272E-05 |
| ENSMUSG00000016427 | Ndufa1 | 0.866 | 0.013393791 |
| ENSMUSG00000017778 | Cox7c | 0.7178 | 0.000450735 |
| ENSMUSG00000020163 | Uqcr11 | 0.6806 | 0.002272881 |
| ENSMUSG00000021290 | Atp5mj | 0.8992 | 1.18223E-05 |
| ENSMUSG00000021606 | Ndufs6 | 0.594 | 0.04043618 |
| ENSMUSG00000022193 | Psmb5 | 0.7168 | 0.020017678 |
| ENSMUSG00000023089 | Ndufa5 | 0.8792 | 1.95619E-05 |
| ENSMUSG00000028410 | Dnaja1 | 0.7668 | 0.032542955 |
| ENSMUSG00000028648 | Ndufs5 | 0.5887 | 0.047108758 |
| ENSMUSG00000028998 | Tomm7 | 0.956 | 0.001450457 |
| ENSMUSG00000029632 | Ndufa4 | 0.536 | 0.041788239 |
| ENSMUSG00000029657 | Hsph1 | 1.5766 | 2.77091E-05 |
| ENSMUSG00000031231 | Cox7b | 0.6621 | 0.003301992 |
| ENSMUSG00000031765 | Mt1 | 2.3292 | 0.000620254 |
| ENSMUSG00000034892 | Rps29 | 1.0966 | 1.75239E-05 |
| ENSMUSG00000035048 | Anapc13 | 0.6413 | 0.041887151 |
| ENSMUSG00000035674 | Ndufa3 | 1.0918 | 2.79884E-06 |
| ENSMUSG00000035885 | Cox8a | 0.5028 | 0.040051897 |
| ENSMUSG00000036216 | Leap2 | 1.2794 | 0.023433913 |
| ENSMUSG00000036372 | Tmem258 | 0.7714 | 0.03233297 |
| ENSMUSG00000037145 | Lypd8l | 1.7976 | 0.000342399 |
| ENSMUSG00000038690 | Atp5mf | 0.7936 | 0.001064168 |
| ENSMUSG00000038717 | Atp5mg | 0.6134 | 0.033874229 |
| ENSMUSG00000039217 | Il18 | 0.6613 | 0.00871162 |
| ENSMUSG00000044894 | Uqcrq | 0.553 | 0.016879759 |
| ENSMUSG00000050856 | Atp5me | 1.1727 | 5.23907E-06 |
| ENSMUSG00000051116 | Gm8121 | 1.4042 | 0.040121937 |
| ENSMUSG00000054422 | Fabp1 | 1.1864 | 0.016930442 |
| ENSMUSG00000057322 | Rpl38 | 1.3131 | 5.93389E-06 |
| ENSMUSG00000059534 | Uqcr10 | 0.7987 | 7.41846E-05 |
| ENSMUSG00000061518 | Cox5b | 0.6713 | 0.005349188 |
| ENSMUSG00000064348 | mt-Tn | 2.0718 | 0.01183543 |
| ENSMUSG00000064349 | mt-Tc | 1.9226 | 0.002272881 |
| ENSMUSG00000064365 | mt-Ts2 | 1.7503 | 0.01662808 |
| ENSMUSG00000064366 | mt-Tl2 | 1.9245 | 2.41101E-09 |
| ENSMUSG00000067288 | Rps28 | 1.2042 | 5.23907E-06 |
| ENSMUSG00000067847 | Romo1 | 0.7348 | 0.042564615 |
| ENSMUSG00000068706 | Gm10250 | 0.681 | 0.045287949 |
| ENSMUSG00000071052 | Rpl7a-ps5 | 1.3342 | 1.71969E-05 |
| ENSMUSG00000071528 | Atp5mk | 1.0127 | 5.23907E-06 |
| ENSMUSG00000073616 | Cops9 | 0.7137 | 0.04043618 |
| ENSMUSG00000074218 | Cox7a1 | 1.125 | 0.001466811 |
| ENSMUSG00000078974 | Sec61g | 0.7868 | 0.042175241 |
| ENSMUSG00000079941 | Cox5b-ps | 0.9183 | 0.002440486 |
| ENSMUSG00000080893 | Ndufa12-ps | 0.9038 | 9.49441E-05 |
| ENSMUSG00000080921 | Rpl38-ps2 | 0.7942 | 0.025934954 |
| ENSMUSG00000081344 | Gm14303 | 0.9024 | 0.002290898 |
| ENSMUSG00000084830 | Gm14539 | 1.6171 | 8.30817E-05 |
| ENSMUSG00000100007 | Gm5527 | 1.0827 | 0.0382235 |
| ENSMUSG00000106037 | Gm4332 | 1.1536 | 0.010652781 |
| ENSMUSG00000106755 | Tpi-rs11 | 1.2191 | 0.025934954 |
| ENSMUSG00000113061 | Rps18-ps5 | 0.9999 | 0.005094121 |
| id | Gene name | Log_2_ Fold Change | FDR |
| ENSMUSG00000113902 | *Ndufb1* | 1.0185 | 0.001466811 |
| ENSMUSG00000115584 | *Gm5854* | 0.9561 | 0.02202619 |
| ENSMUSG00000117621 | *Hspe1-rs1* | 1.2641 | 0.000450735 |
| ENSMUSG00000120390 | *Gm56743* | 1.5247 | 6.63621E-05 |
