# Supplementary material for: Lactiplantibacillus plantarum GUANKE alleviates Zearalenone-induced intestinal dysfunction by modulating oxidative stress and inflammation
Source: PLoS One. 2026 Jul 1;21(7):e0351300. doi: 10.1371/journal.pone.0351300 (PMC13322542; doi:10.1371/journal.pone.0351300)
Supplement: S6 Table — (DOCX) [file pone.0351300.s007.docx]

**S6 Table.**  **Downregulated differentially expressed genes (DEGs) in GK group vs. ZEN group**

| id | Gene name | Log_2_ Fold Change | FDR |
| --- | --- | --- | --- |
| ENSMUSG00000013523 | *Bcas1* | -1.667 | 0.041788239 |
| ENSMUSG00000017607 | *Tns4* | -1.0572 | 0.036899253 |
| ENSMUSG00000020695 | *Mrc2* | -2.2074 | 0.003452899 |
| ENSMUSG00000020787 | *P2rx1* | -1.9619 | 0.016879759 |
| ENSMUSG00000024421 | *Lama3* | -2.3595 | 0.01820925 |
| ENSMUSG00000024621 | *Csf1r* | -1.1725 | 0.041788239 |
| ENSMUSG00000025002 | *Cyp2c55* | -2.3364 | 0.001450457 |
| ENSMUSG00000025810 | *Nrp1* | -1.8549 | 0.046156543 |
| ENSMUSG00000026768 | *Itga8* | -2.0973 | 0.042175241 |
| ENSMUSG00000026837 | *Col5a1* | -1.7436 | 0.025627375 |
| ENSMUSG00000027009 | *Itga4* | -1.3956 | 0.040168296 |
| ENSMUSG00000028369 | *Svep1* | -2.0187 | 0.032542955 |
| ENSMUSG00000029298 | *Gbp9* | -1.4608 | 0.041788239 |
| ENSMUSG00000029490 | *Slc49a3* | -1.493 | 0.000456812 |
| ENSMUSG00000030866 | *Ern2* | -1.4007 | 0.004475928 |
| ENSMUSG00000031740 | *Mmp2* | -2.4649 | 0.041788239 |
| ENSMUSG00000031981 | *Capn9* | -1.9557 | 0.013701577 |
| ENSMUSG00000034684 | *Sema3f* | -1.6026 | 0.019162361 |
| ENSMUSG00000036862 | *Dchs1* | -1.7768 | 0.039529066 |
| ENSMUSG00000037106 | *Fer1l6* | -1.8231 | 0.03233297 |
| ENSMUSG00000041828 | *Abca8a* | -1.9589 | 0.000456812 |
| ENSMUSG00000044338 | *Aplnr* | -1.9121 | 0.016879759 |
| ENSMUSG00000046805 | *Mpeg1* | -1.2952 | 0.033874229 |
| ENSMUSG00000047562 | *Mmp10* | -2.4531 | 0.010612555 |
| ENSMUSG00000057657 | *Rps18-ps3* | -1.4955 | 0.002092481 |
| ENSMUSG00000059588 | *Calcrl* | -1.8047 | 0.007243714 |
| ENSMUSG00000063415 | *Cyp26b1* | -2.484 | 5.23907E-06 |
| ENSMUSG00000067229 | *Cyp2c66* | -1.2797 | 0.014257355 |
| ENSMUSG00000083087 | *Gm11249* | -2.525 | 0.032542955 |
| ENSMUSG00000095351 | *Igkv3-2* | -2.4223 | 0.041887151 |
| ENSMUSG00000116972 | *Gm6278* | -2.1085 | 0.00871162 |
