# Supplementary material for: Lactiplantibacillus plantarum GUANKE alleviates Zearalenone-induced intestinal dysfunction by modulating oxidative stress and inflammation
Source: PLoS One. 2026 Jul 1;21(7):e0351300. doi: 10.1371/journal.pone.0351300 (PMC13322542; doi:10.1371/journal.pone.0351300)
Supplement: S7 Table — (DOCX) [file pone.0351300.s008.docx]

**S7 Table. GSEA enrichment analysis**

|  | Cont. vs ZEN | | ZEN vs GK | |  |  |
| --- | --- | --- | --- | --- | --- | --- |
| Intersect(p<0.05) | P-value | NES | P-value | NES | NES-sum | P-value-sum |
| GOBP_ADAPTIVE_IMMUNE_RESPONSE | 0.000163613 | 1.7125757 | 0.0007145 | -1.348674 | 3.061250089 | 0.000878153 |
| GOBP_REGULATION_OF_LYMPHOCYTE_ACTIVATION | 0.000164447 | 1.6637766 | 0.0013199 | -1.332134 | 2.995910465 | 0.001484341 |
| GOBP_LYMPHOCYTE_ACTIVATION | 0.00015949 | 1.5726317 | 0.0001140 | -1.363507 | 2.936138693 | 0.000273463 |
| GOBP_ADAPTIVE_IMMUNE_RESPONSE_BASED_ON_SOMATIC_RECOMBINATION_OF_IMMUNE_RECEPTORS_BUILT_FROM_IMMUNOGLOBULIN_SUPERFAMILY_DOMAINS | 0.000168776 | 1.6657083 | 0.0189610 | -1.257826 | 2.923534611 | 0.019129717 |
| GOBP_POSITIVE_REGULATION_OF_IMMUNE_RESPONSE | 0.000163613 | 1.5558552 | 0.0009457 | -1.317004 | 2.872863697 | 0.001109351 |
| GOBP_REGULATION_OF_NIK_NF_KAPPAB_SIGNALING | 0.008378871 | 1.4583233 | 0.0364760 | -1.336838 | 2.795161554 | 0.044854823 |
| GOBP_IMMUNE_EFFECTOR_PROCESS | 0.000162285 | 1.5039063 | 0.0017409 | -1.288434 | 2.792340623 | 0.001903232 |
| GOBP_POSITIVE_REGULATION_OF_IMMUNE_SYSTEM_PROCESS | 0.000157803 | 1.4395355 | 0.0002229 | -1.309465 | 2.74900028 | 0.000380719 |
| GOBP_LEUKOCYTE_CELL_CELL_ADHESION | 0.003377237 | 1.3312706 | 0.0016313 | -1.365087 | 2.696357974 | 0.005008559 |
| GOBP_REGULATION_OF_IMMUNE_RESPONSE | 0.000158629 | 1.4184252 | 0.0011224 | -1.262604 | 2.681029095 | 0.00128109 |
| GOBP_POSITIVE_REGULATION_OF_LEUKOCYTE_MIGRATION | 0.047908473 | 1.2717086 | 0.0078336 | -1.407525 | 2.679233492 | 0.055742115 |
| GOBP_T_CELL_ACTIVATION | 0.002975698 | 1.3047837 | 0.0007268 | -1.353949 | 2.658733095 | 0.003702531 |
| GOBP_REGULATION_OF_T_CELL_ACTIVATION | 0.003199192 | 1.3451555 | 0.0104512 | -1.297516 | 2.642671062 | 0.013650377 |
| GOBP_REGULATION_OF_IMMUNE_SYSTEM_PROCESS | 0.000152207 | 1.3540526 | 0.0001066 | -1.277288 | 2.631340488 | 0.000258771 |
| GOBP_IMMUNE_RESPONSE | 0.000150083 | 1.3445537 | 0.0001051 | -1.279263 | 2.62381645 | 0.000255169 |
| GOBP_LEUKOCYTE_MIGRATION | 0.023632681 | 1.2260393 | 0.0033839 | -1.339769 | 2.565807884 | 0.027016563 |
| GOBP_NIK_NF_KAPPAB_SIGNALING | 0.046028881 | 1.2800498 | 0.0477419 | -1.284283 | 2.564333233 | 0.093770816 |
| GOBP_MONONUCLEAR_CELL_DIFFERENTIATION | 0.030489837 | 1.1959311 | 0.0025738 | -1.331198 | 2.527129043 | 0.033063682 |
| GOBP_ERK1_AND_ERK2_CASCADE | 0.042917727 | 1.2084760 | 0.0078985 | -1.312969 | 2.521445199 | 0.050816212 |
| GOBP_RESPONSE_TO_BACTERIUM | 0.002761982 | 1.2578447 | 0.0349391 | -1.178249 | 2.436094102 | 0.037701042 |
